# Improving the Stability of Maleimide–Thiol Conjugation for Drug Targeting

**DOI:** 10.1002/chem.202003951

**Published:** 2020-10-27

**Authors:** Marianne Lahnsteiner, Alexander Kastner, Josef Mayr, Alexander Roller, Bernhard K. Keppler, Christian R. Kowol

**Affiliations:** ^1^ Faculty of Chemistry Institute of Inorganic Chemistry University of Vienna Waehringer Strasse 42 1090 Vienna Austria; ^2^ Research Cluster „Translational Cancer Therapy Research“ Waehringer Strasse 42 1090 Vienna Austria

**Keywords:** bioconjugation, bioorganic chemistry, drug delivery, maleimide, retro-Michael reactions

## Abstract

Maleimides are essential compounds for drug conjugation reactions via thiols to antibodies, peptides and other targeting units. However, one main drawback is the occurrence of thiol exchange reactions with, for example, glutathione resulting in loss of the targeting ability. A new strategy to overcome such retro‐Michael exchange processes of maleimide–thiol conjugates by stabilization of the thiosuccinimide via a transcyclization reaction is presented. This reaction enables the straightforward synthesis of stable maleimide–thiol adducts essential in drug‐conjugation applications.

## Graphical abstract

**An** ***in situ*** **transcyclization** serves as a stabilizing method for the commonly used maleimide–thiol conjugation. This prevents subsequent retro‐Michael reactions for example, with glutathione (see scheme).

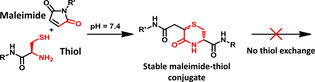

In the past 50 years, the use of maleimide compounds as Michael acceptors has become a common way for conjugation to thiol‐bearing molecules.^[1]^ The applications vary from peptide‐ and antibody‐drug conjugates, fluorescent‐labeling of biomolecules as well as PEGylation of peptides and proteins. For example, in 2011 brentuximab vedotin was approved by the FDA for the treatment of Hodgkin lymphoma where the highly cytotoxic antimitotic agent monomethyl auristation E is conjugated via a maleimide moiety to a cysteine of the CD30‐specific antibody.^[2]^ Also, in the case of trastuzumab emtansine, approved for metastatic breast cancer, a maleimide moiety is used. In this case, the maleimide is attached to the antibody and reacts with a thiol group of the cytotoxic drug.^[3]^ In general, the use of maleimides has many advantages, like fast kinetics, quantitative conversion and high specificity. Nevertheless, the crucial factors for successful drug delivery, namely stability of the conjugate and controlled release, are not yet fully provided. The main weakness is a possible thiol exchange (e.g. with glutathione; GSH) of the formed thiosuccinimide, induced by a retro‐Michael reaction. This β‐elimination reaction results in the loss of targeting properties and, therefore, promotes off‐target activity.^[4]^ One of the few possibilities to diminish this problem of maleimides is to exploit the fast hydrolysis of thiosuccinimides when electron‐withdrawing moieties are present, resulting in the formation of stable thioethers (thiosuccinimides).^[5]^ However, this strategy is limited to *N*‐aryl substituted maleimides[^5a^, ^6^] or other electron‐withdrawing *N*‐substituents.^[7]^ The much more commonly used *N*‐alkyl‐substituted derivatives show too slow hydrolysis of the formed thiol adducts to generate the desired stable thioethers.

In a recent publication, we synthesized a drug–peptide conjugate via reaction of a maleimide moiety and an *N*‐terminal cysteine for coupling.^[8]^ High‐performance liquid chromatography/mass spectrometry (HPLC‐MS) measurements revealed that the product peak converted within several hours into a new peak with the same exact mass. This reaction was supposed to be a Michael‐transcyclization already known from similar systems.^[9]^ (Figure 1).

**Figure 1:**
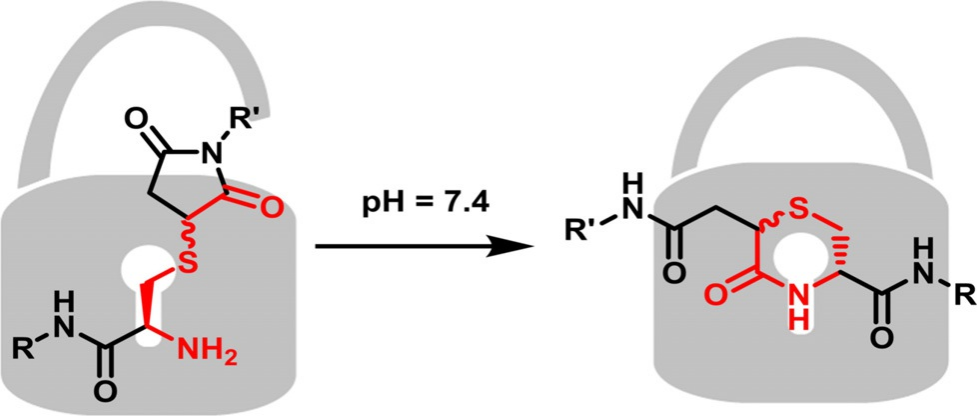
Locking the thioether conjugation bond in a 6‐membered ring via a transcyclization reaction.

We assume that the formation of the six‐membered ring is locking the thioether conjugation moiety and the transcyclization reaction is, therefore, an elegant method to prevent retro‐Michael reactions. Consequently, this strategy serves as a promising tool for the synthesis of stable maleimide–thiol drug conjugates.

Herein, we report on the detailed analysis of the postulated transcyclization reaction using a model compound system by HPLC‐MS measurements and investigated the stability in the presence of GSH. Furthermore, the feasibility of this method was confirmed for a drug‐peptide conjugate.

As a first step, a model reaction to study and prove the suggested transcyclization reaction was developed (Figure 2). Therefore, l‐cysteine methyl ester hydrochloride (**1a**) was reacted with *N*‐ethylmaleimide (**2**) in phosphate buffer (PB) at pH 7.4 (Figure 3 A). As anticipated, the instantly formed maleimide‐thiol conjugate **3a** (*t*=0 h, rt=4.3 and 5.3 min) underwent a conversion reaction, leading to a more hydrophobic compound with the same mass (*m*/*z=*261) with a retention time of 9.1 min (Figure 3 A) assigned to the transcyclization product **4** (Figure 2 A). The fact that the initial Michael adduct **3a** appeared as two separate peaks in the extracted ion chromatogram (EIC) can be explained by the formation of diastereomers. In contrast, in case of **4** only one peak was observed (Figure 3 A). When the HPLC conditions were adjusted to a flatter gradient, a second isomer could be observed for **4** as well, however, with just ≈10 % abundancy (Figure S1). This imbalance can probably be explained by steric hindrance of the substituents of the thiomorpholinone core. The ratio between the two diastereomers of **3a** did not change over time revealing no preference for one diastereomer in the transcyclization process. The negative control reaction was performed with *N*‐acetyl l‐cysteine methyl ester (**1b**) and *N*‐ethyl maleimide (**2**) where the transcyclization is supposedly prevented by the protected amino moiety (Figure 2 B). Incubation of the compounds under the same conditions resulted in the instant formation of only one peak (rt=10.2 min, *m*/*z=*303) attributed to the thiosuccinimide **3b** which was stable for more than 24 h (Figure 3 B; even with a less steep gradient, the second diastereomer could not be observed. However, two sets of peaks are present in the NMR spectra; see ESI). Consequently, as desired, the protection of the amino moiety prevented the transcyclization reaction.

**Figure 2:**
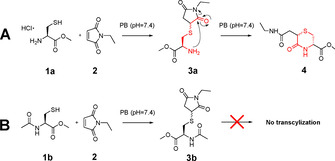
**A**. Model system for the transcyclization reaction using l‐cysteine methyl ester hydrochloride as a Michael donor and *N*‐ethyl maleimide as Michael acceptor. **B**. Reaction of *N*‐acetyl protected l‐cysteine methyl ester with *N*‐ethyl maleimide served as negative control.

**Figure 3:**
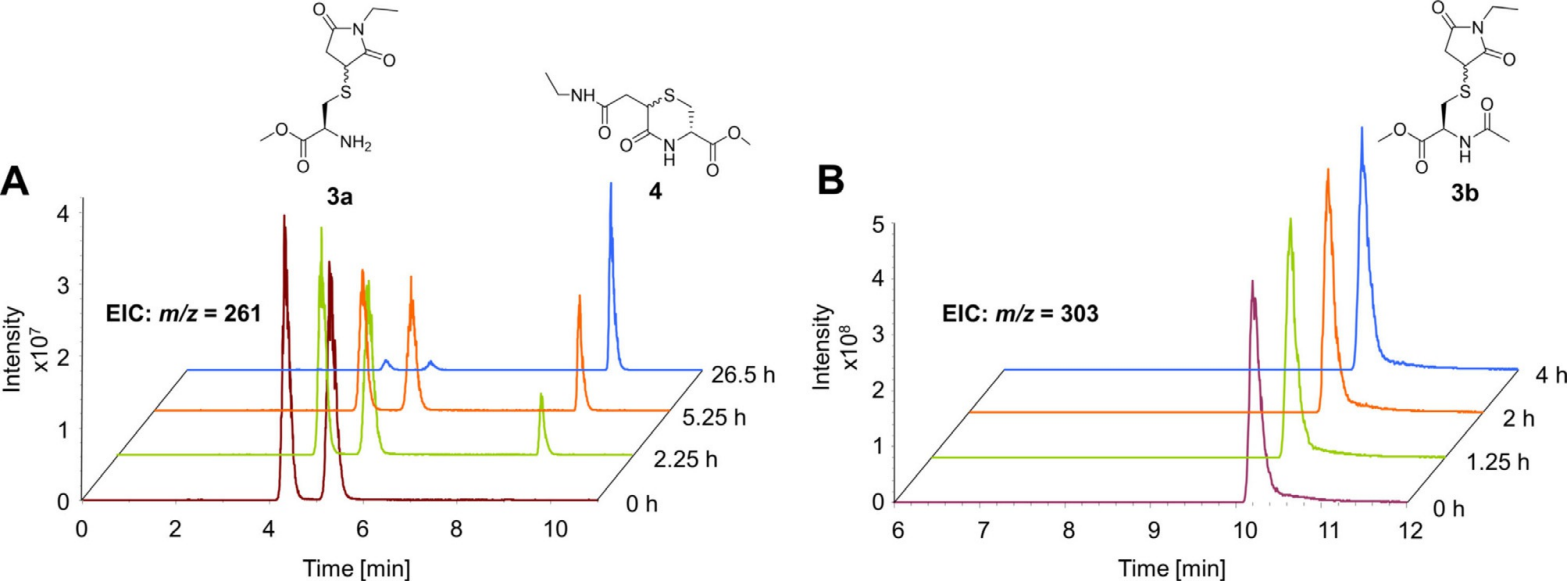
HPLC‐MS EIC traces. **A**. Reaction of cysteine methyl ester hydrochloride (**1a**) and *N*‐ethylmaleimide (**2**). Immediate formation of the Michael adduct diastereomers (**3a**) (*m*/*z=*261, rt=4.4 and 5.4 min) and time‐dependent transcyclization into **4** (*m*/*z=*261, rt=9.1 min). **B**. Reaction of *N*‐acetyl cysteine methyl ester (**1b**) with *N*‐ethylmaleimide (**2**) with immediate formation of the stable Michael adduct (**3b**) (*m*/*z=*303, rt=10.2 min), without subsequent transcyclization. Both reactions were performed at 50 μm of each reactant in phosphate buffered solution at pH 7.4 and 25 °C.

Since the Michael adduct (**3a**) and the transcyclization product (**4**) possess the same exact mass, we also synthesized the compounds (together with **3b**) and characterized them in detail via ^1^H and ^13^C NMR spectroscopy to confirm their chemical identity. Compound **3a** was generated from **1a** and **2** in MeOH to keep the free amino group protonated and avoid the ring closing reaction. In contrast, **4** was synthesized from the same educts, however in 100 mm PB solution at pH 7.4. The NMR of **4** clearly proofed the transcyclization reaction: On the one hand the absence of an amine NH_2_ signal, but the presence of two NH signals (8.07 and 7.89 ppm). On the other hand, a cross peak in the heteronuclear multiple bond correlation (HMBC) spectrum of the proton located at N2 (8.07 ppm) and C6 (186.07 ppm), in line with the newly formed bond. The presence of diastereomers in case of **3a** was indicated by two ^13^C signals for each carbon atom. Notably, the retention time of both synthesized compounds in the HPLC perfectly fitted to the co‐incubation experiments in Figure 3, confirming the peak assignment. For the purified transcyclization product **4** a X‐ray single crystal structure^[18a]^ could be obtained (Figure 4; Table S1–S3). The crystal structure showed one diastereomer^[18b]^ namely the *trans*‐oriented conformations at C5 (*S*) and C7 (*R*). Notably, in the literature only the *cis*‐conformer was reported in the case of *N*‐phenyl maleimide and l‐cysteine methyl ester.^[9a]^ However, the absence of a ^1^H–^1^H‐NOESY‐cross peak between C5 and C7 in the NMR spectrum of **4** (which is clearly visible for the *cis*‐isomer^7^) suggests the presence of the *trans* isomer also in solution.

**Figure 4:**
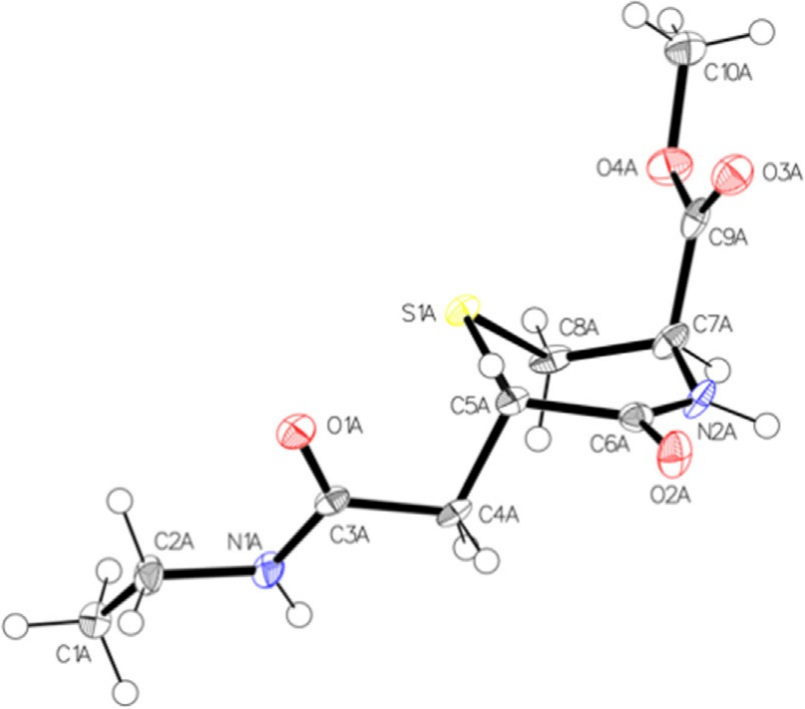
Crystal structure of the transcyclization product **4**, drawn with 50 % displacement ellipsoids. For clarity only one of three independent molecules in the asymmetric unit is displayed. The stereocenters at C5 and C7 are *trans* configured.

To prove the superior stability of a transcyclization product under retro‐Michael conditions we performed co‐incubation experiments with GSH. On the one hand, we compared the stability of the thiosuccinimide **3b** and the transcyclization product **4**. On the other hand we synthesized two peptide‐drug conjugates as „real world“ examples. We used the epidermal growth factor receptor (EGFR)‐binding peptide (Leu‐Ala‐Arg‐Leu‐Leu‐Thr; LARLLT) and an oxaliplatin(IV)‐maleimide complex (Figure 5). For conjugation a Cys‐miniPEG linker was attached to the LARLLT peptide sequence. We synthesized the transcyclization product **5a** using 24 h incubation of the peptide and the maleimide‐bearing platinum complex in PB pH 7.4,^[8]^ resulting in ∼95 % conversion to **5a**. For the thiosuccinimide reference complex **5b** we protected the terminal Cys via acetylation. All compounds (50 μm) were incubated in an aqueous phosphate buffered solution (100 mm, pH 7.4, 25 °C) in the presence of 10‐fold excess of reduced GSH for 25 h and the reaction was monitored via HPLC‐MS. The *N*‐acetylated, open‐chain complex **5b** underwent distinct thiol‐exchange reaction with GSH (Figure 5 A) with formation of the oxaliplatin(IV)‐thiosuccinimide‐GSH species at *m*/*z=*947. In contrast, the transcyclization bioconjugate **5a** did not show significant GSH‐adduct formation even after 25 h (Figure 5 B). The same picture could be observed for the model compounds: **4** was basically not affected by the presence of GSH, whereas in case of **3b** the *N*‐ethylthiosuccinimide‐GSH adduct was formed (Figure S2). The conversion to the GSH adduct proceeded with ∼0.5 % h^−1^ resulting in ∼15 % after 25 h incubation.

**Figure 5:**
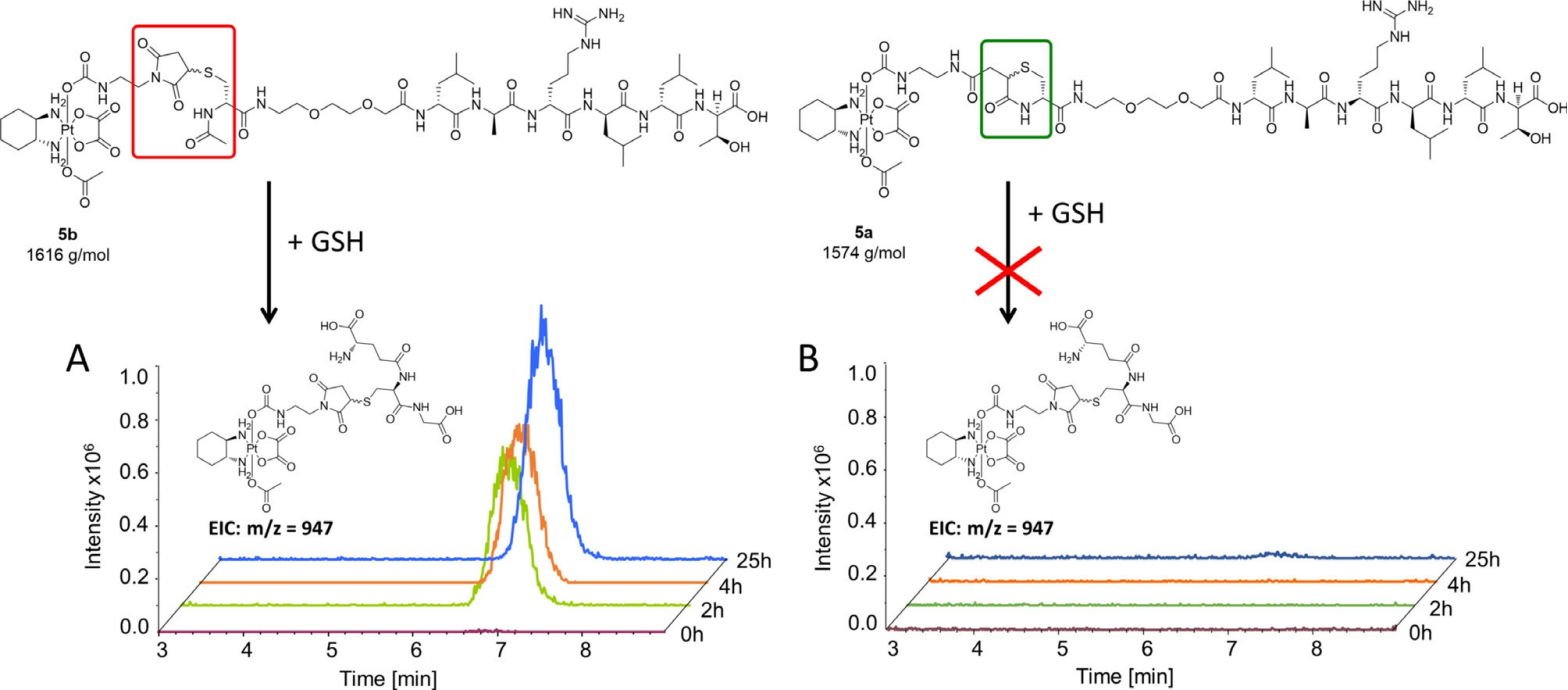
HPLC‐MS EIC traces of the oxaliplatin(IV)‐thiosuccinimide‐GSH adduct (*m*/*z=*947, rt=6.8 min). Incubation of 50 μm **5a** (**A**) or **5b** (**B**) with a 10‐fold excess of GSH in PB (100 mm, pH 7.4) at 25 °C over the course of 25 h.

Taken together, this data shows that in the presence of a cysteine moiety maleimide–thiol bonds can be stabilized against a retro‐Michael thiol exchange reaction with only minimal additional effort. The respective transcyclization can be achieved simply by an extended incubation time in buffered solution. In the last years a lot of work was put into the discovery of SH conjugation moieties other than maleimides, which also increase the stability against retro‐Michael reactions. For example *exo*‐cyclic maleimides,^[10]^ sulfones,^[11]^ carbonylacrylic reagents^[12]^ or 2‐formylphenylboronic acids^[13]^ (Figure S3). Fact is, however, that most of the thiol‐coupling reagents commercially available are still common (alkyl) maleimides: for example, succinimidyl‐4‐(*N*‐maleimidomethyl)cyclohexane‐1‐carboxylate (SMCC) is one of the most popular linkers for antibody coupling, *N*‐(γ‐maleimidobutyryloxy)succinimide ester (GMBS) or similar derivatives are used for peptide coupling and dibenzoazacyclooctyne‐maleimide (Mal‐DBCO) for click chemistry or crosslinking reagents with two or more maleimide moieties.^[14]^ Therefore, a method which still uses maleimides, but strongly enhances the stability, is of high interest. Different types of application for the new method can be distinguished: 1) attachment of an *N*‐terminal cysteine to any peptide for (drug) conjugation and subsequent reaction with the desired maleimide. 2) Peptides which already contain a cysteine, but where the incubation time probably was not sufficient to generate the stabilized transcyclization product^[15]^ or the terminal cysteine amino group was protected.^[16]^ 3) Targeted (drug) conjugates where currently alkylthiols are used for maleimide coupling^[17]^ and 4) antibody‐drug conjugates where the SMCC maleimide linker is attached to a lysine of the antibody and the drug is modified with a cysteine linker to enable transcyclization.

## Conflict of interest

The authors declare no conflict of interest.

## Supporting information

As a service to our authors and readers, this journal provides supporting information supplied by the authors. Such materials are peer reviewed and may be re‐organized for online delivery, but are not copy‐edited or typeset. Technical support issues arising from supporting information (other than missing files) should be addressed to the authors.

Supplementary
