# Supplementary material for: Improving the Stability of Maleimide–Thiol Conjugation for Drug Targeting
Source: Chemistry. 2020 Oct 27;26(68):15867–70. doi: 10.1002/chem.202003951 (PMC7756610; doi:10.1002/chem.202003951)
Supplement: Supplementary file 1 — Supplementary [file CHEM-26-15867-s001.pdf]

Chemistry–A European Journal

Supporting Information

Improving the Stability of Maleimide–Thiol Conjugation for Drug Targeting

Marianne Lahnsteiner+,[a] Alexander Kastner+,[a] Josef Mayr,[a] Alexander Roller,[a] Bernhard K. Keppler,[a, b] and Christian R. Kowol*[a, b]

Table of contents

Materials and methods

Synthesis and characterization of compounds

Experimental details for X-ray diffraction and HPLC-MS

Figures and tables

References

Materials and methods

All solvents and reagents were purchased from commercial suppliers and were used without further purification. Anhydrous THF, DMF, toluene and acetonitrile were bought from Sigma-Aldrich over molecular sieves. For all reactions with platinum, the water used was taken from the osmosis plant and further distilled twice. Cys-mini-PEG-LARLLT and N-Acetyl-Cys-mini-PEG-LARLLT was ordered from Biomatik. All other chemicals were bought from Sigma Aldrich, Acros, Fisher, TCI or Alfa Aesar. (*OC*-6-33)-[(1*R*,2*R*)-cyclohexane-1,2-diamine](2-(2,5-dioxo-2,5-dihydro-1*H*-pyrrol-1-yl)ethylcarbamato)oxalato platinum(IV) was synthesized according to literature[1]. Electrospray ionization mass spectra (ESI-MS) were performed with a Bruker Esquire$_{3000}$ ion trap spectrometer. A Bruker FT-NMR Avance III 500 MHz spectrometer at 600.25 (^{1}H) and 150.93 (^{13}C) respectively, was used for recording NMR spectra. DMSO-d_6 was used as solvent. Chemical shifts (ppm) were referenced internally to the solvent residual peaks. Purification with preparative RP-HPLC was carried out with an Agilent 1200 Series system controlled by Chemstation software. For all HPLC-MS measurements an Agilent 1260 Infinity system was used. The MS part was performed on a Bruker AmaZon SL electrospray ionization ion trap mass spectrometry system. The experimental conditions were set as follows: positive ionization mode; drying gas flow: 10 l/min (350°C); nebulizer pressure: 35 psi; capillary voltage: 4000 V. Data evaluation and instrument control was carried out with HyStar 3.2 and Data Analysis 4.0 software package (Bruker Daltonics).

Syntheses and characterization

Methyl *S*-(1-ethyl-2,5-dioxopyrrolidin-3-yl)-L-cysteinate (3a)

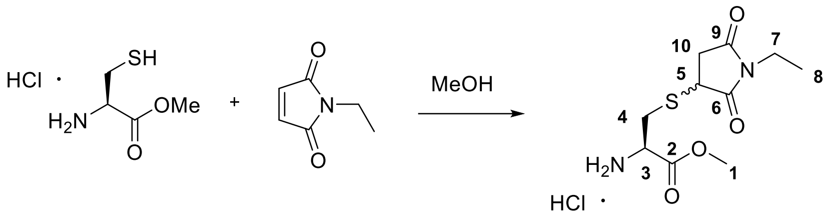

N-Ethylmaleimide (73 mg, 0.582 mmol) and L-cysteine methyl ester hydrochloride (100 mg, 0.582 mmol) were dissolved in 25 mL MeOH. The resulting reaction mixture was stirred at room temperature for 20 min and the solvent was evaporated *in vacuo.* Purification was performed via preparative HPLC (C_{18} X-Bridge, H_2O/ACN (both with 0.1% formic acid), isocratic with 10% ACN). Yield: 115 mg (76%) in two fractions, one for each diastereomer. MS: calcd. for $[C_{10}H_{16}N_2O_4S+Na^+]^+$ = 261.0904, found: 261.0906.

First diastereomer: ^{1}H NMR (DMSO-d_6): δ 8.54 (s, 3H, NH_3), 4.43 (t, J = 6.1 Hz, 1H, C3H), 4.04 (dd, J = 9.1, 4.2 Hz, 1H, C5H), 3.78 (s, 3H, C1H_3), 3.42 (q, J = 7.1 Hz, 2H, C7H_2), 3.26 (dd, J = 14.1, 6.2 Hz, 2H, C4H_2), 3.18 (dd, J = 18.3, 9.1 Hz, 1H, C10H), 2.52 (m, 1H, C10H), 1.05 (t, J = 7.1 Hz, 3H, C8H_3) ppm; ^{13}C NMR (DMSO-d_6): δ 176.78 (C6/9), 174.53 (C6/9), 168.47 (C2), 53.17 (C1), 51.96 (C3), 39.95 (C5), 35.47 (C10), 33.34 (C7), 31.37 (C4), 12.62 (C8) ppm.

Second diastereomer: ^{1}H NMR (DMSO-d_6): δ 8.57 (s, 3H, NH_3), 4.42 (t, J = 6.3 Hz, 1H, C3H), 4.06 (dd, J = 8.8, 3.9 Hz, 1H, C5H), 3.78 (s, 3H, C1H_3), 3.41 (q, J = 7.2 Hz, 2H, C7H_2), 3.16 (dd, J = 18.1, 9.2 Hz, 2H, C4H_2), 3.09 (dd, J = 14.3, 7.1 Hz, 1H, C10H), 2.51 (m, 1H, C10H), 1.06 (t, J = 7.1Hz, 3H, C8H_3) ppm; ^{13}C NMR (DMSO-d_6): δ 176.53 (C6/9), 174.61 (C6/9), 168.48 (C2), 53.15 (C1), 51.51 (C3), 38.92 (C5), 35.27 (C10), 33.23 (C7) 30.84 (C4), 12.59 (C8) ppm.

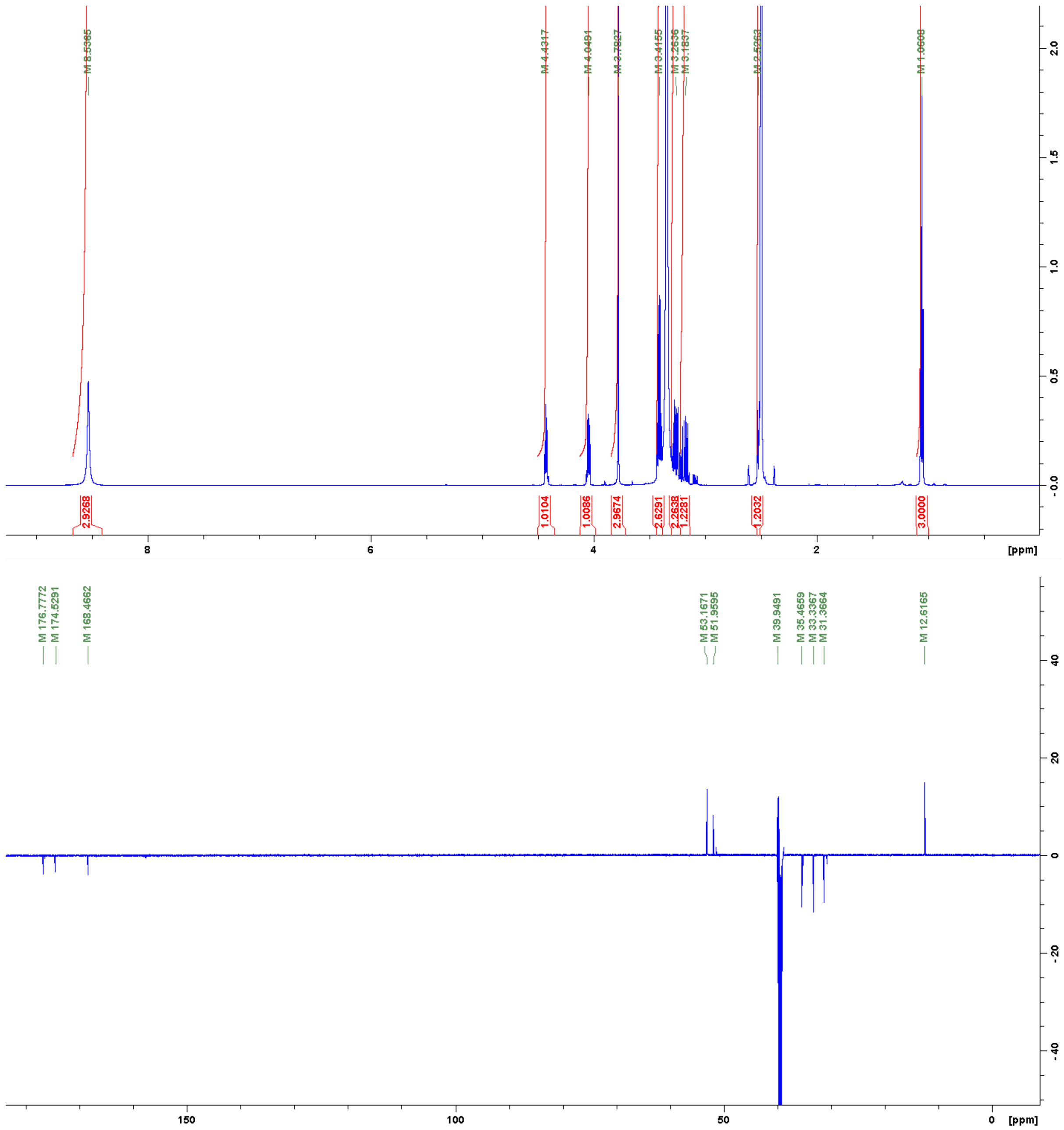

^{1}H and ^{13}C NMR spectra of first diastereomer of **3a** (with traces of the other diastereomer).

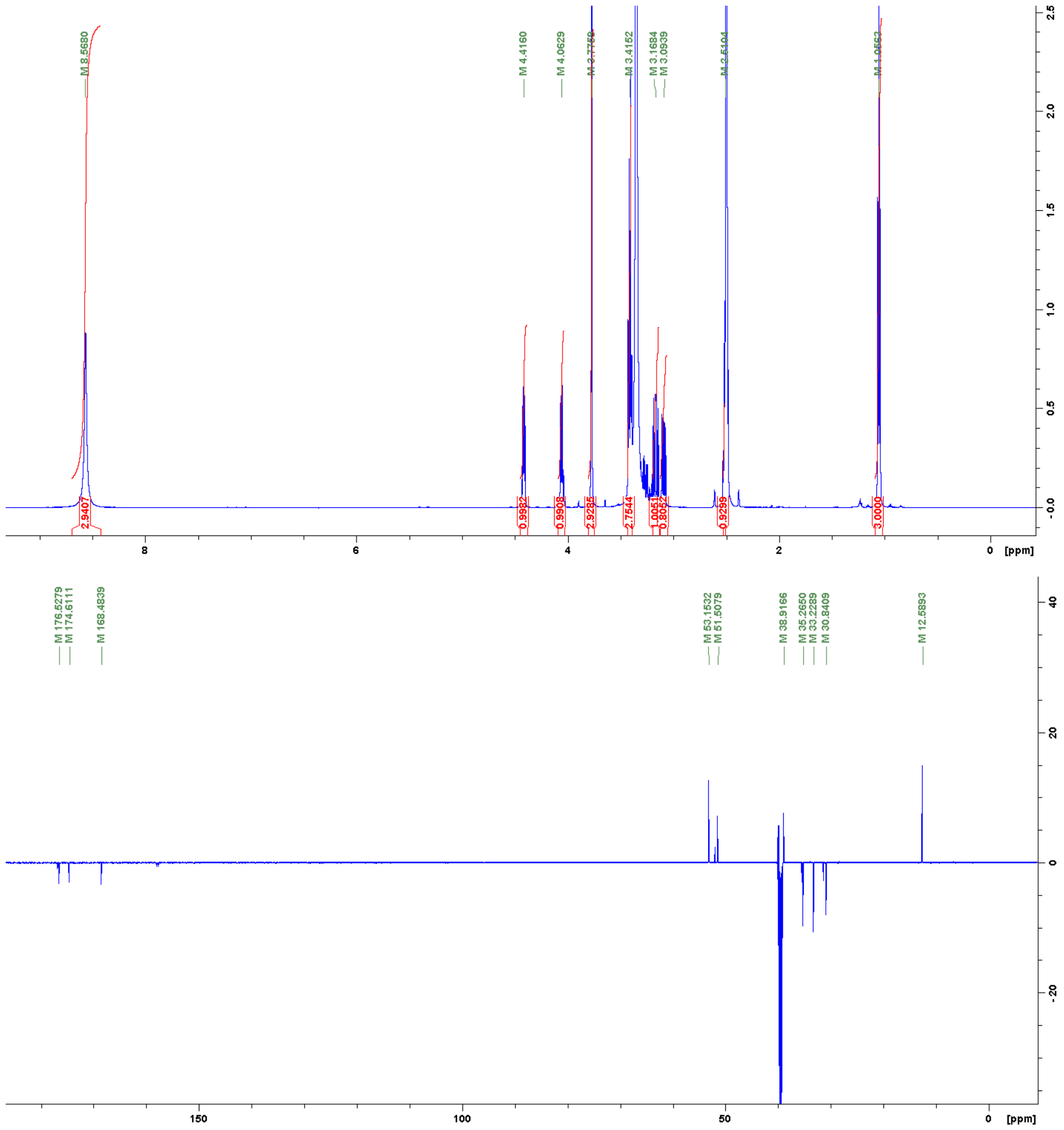

^{1}H and ^{13}C NMR spectra of the second diastereomer of **3a** (with traces of the other diastereomer).

Methyl S-(1-ethyl-2,5-dioxopyrrolidin-3-yl)-L-cysteinate (3b)

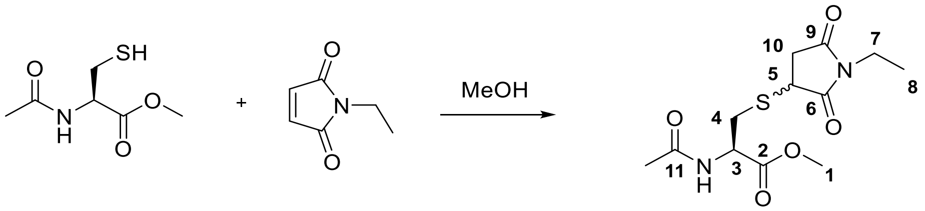

N-Ethylmaleimide (35 mg, 0.282 mmol) was dissolved in 15 mL MeOH and then N-acetyl-L-cysteine methyl ester (50 mg, 0.282 mmol) was added. The resulting reaction mixture was stirred at room temperature for 22 h. The solvent was removed *in vacuo*. Purification was performed via preparative HPLC (C_{18} X-Bridge, H_2O/ACN (both with 0.1% formic acid), isocratic with 15% ACN). Yield: 51 mg (59%). MS: calcd. for $[C_{12}H_{18}N_2O_5S+Na^+]^+$ = 325.0829, found: 325.0832; Only one set of ^{1}H NMR signals could be observed, albeit they are quite broad. ^{1}H NMR (DMSO-d_6): δ 8.45 (m, 1H, N*H*), 4.51 (m, 1H, C3*H*), 3.99 (dd, *J* = 9.0 Hz, 1H, C5*H*), 3.64 (s, 3H, C1H_3), 3.25 (m, 1H, C4*H*), 3.12 (m, 2H, C5H_2), 2.89 (m, 1H, C4*H*), 1.87 (s, 3H, C12*H*), 1.05 (t, *J* = 7.2 Hz, 3H, C8H_3) ppm; Two sets of ^{13}C NMR signals could be observed for almost all peaks, however, they could not be assigned to the respective diastereomers. ^{13}C NMR (DMSO-d_6): δ 176.51/176.44 (C6/9), 174.79/174.72 (C6/9), 171.03/170.99 (C2), 169.49/169.36 (C11), 52.18/52.12 (C1), 51.93/51.48 (C3), 38.90 (C5), 35.75/35.54 (C10), 33.20/33.15 (C7), 32.24/32.06 (C4), 22.26/22.24 (C12), 12.57 (C8) ppm

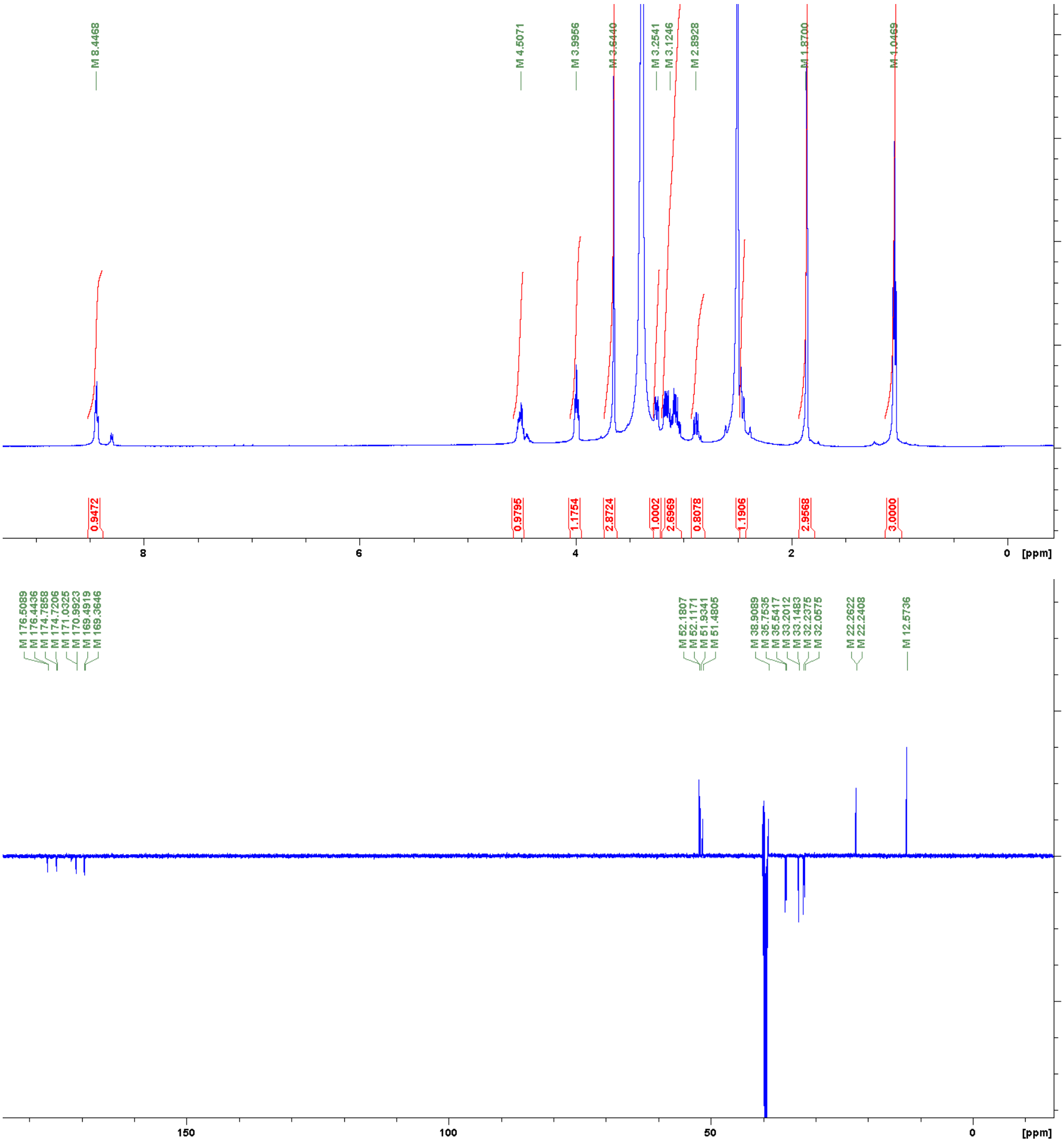

^{1}H and ^{13}C NMR spectra of **3b.**

Methyl (3*R*,6*S*)-6-(2-(ethylamino)-2-oxoethyl)-5-oxothiomorpholine-3-carboxylate (4)

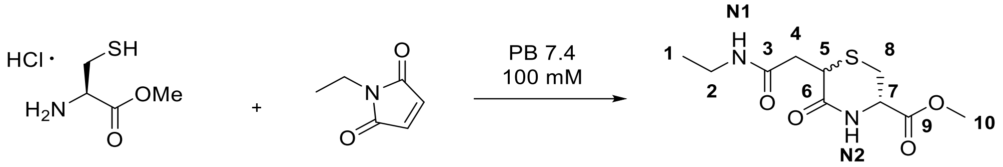

L-cysteine methyl ester hydrochloride (30 mg, 0.175 mmol) was dissolved in 5 ml distilled water and the pH was adjusted to 7.4 with NaOH. Afterwards, 25 ml phosphate buffer (pH = 7.4, 100 mM) were added, as well as *N*-ethylmaleimide (21.3 mg, 0.175 mmol), predissolved in 250 µL DMSO. The reaction mixture was stirred for 24 h and subsequently lyophilized. Purification was performed via preparative HPLC (C_{18} X-Bridge, H_2O/ACN (both with 0.1% formic acid), isocratic with 10% ACN). Yield: 10 mg (20%). During incubation in phosphate buffer a considerable amount of the transcyclisation product is deprotected at the carboxylic acid, which results in such low yields. Furthermore, during preparative HPLC the second diastereomer was separated off. MS: calcd. for $[C_{10}H_{16}N_2O_4S+Na^+]^+$ = 283.0723, found: 283.0726; ^{1}H NMR (DMSO-d_6): δ 8.06 (b, J = 4.5 Hz, 1H, N2*H*), 7.89 (b, 1H, N1*H*), 4.45 (q, J = 4.4 Hz, 1H, C7*H*), 3.70 (s, 3H, C10H_3), 3.59 (dd, J = 9.5, 4.2 Hz, 1H, C5*H*), 3.15 (dd, J = 13.6, 4.6 Hz, 1H, C8*H*), 3.06 (m, 2H, C2H_2), 2.97 (dd, J = 13.6, 4.4 Hz, 1H, C8*H*), 2.69 (dd, J = 14.9, 4.2 Hz, 1H, C4*H*), 2.46 (dd, J = 14.9, 9.5 Hz, 1H, C4*H*), 1.00 (dd, J = 9.2, 5.3 Hz, 3H, C1H_3) ppm; ^{13}C NMR (DMSO-d_6): δ 170.99 (C9), 168.80 (C3), 168.07 (C6), 55.96 (C7), 52.52 (C10), 38.40 (C4), 37.34 (C5), 33.45 (C2), 25.42 (C8), 14.68 (C1) ppm.

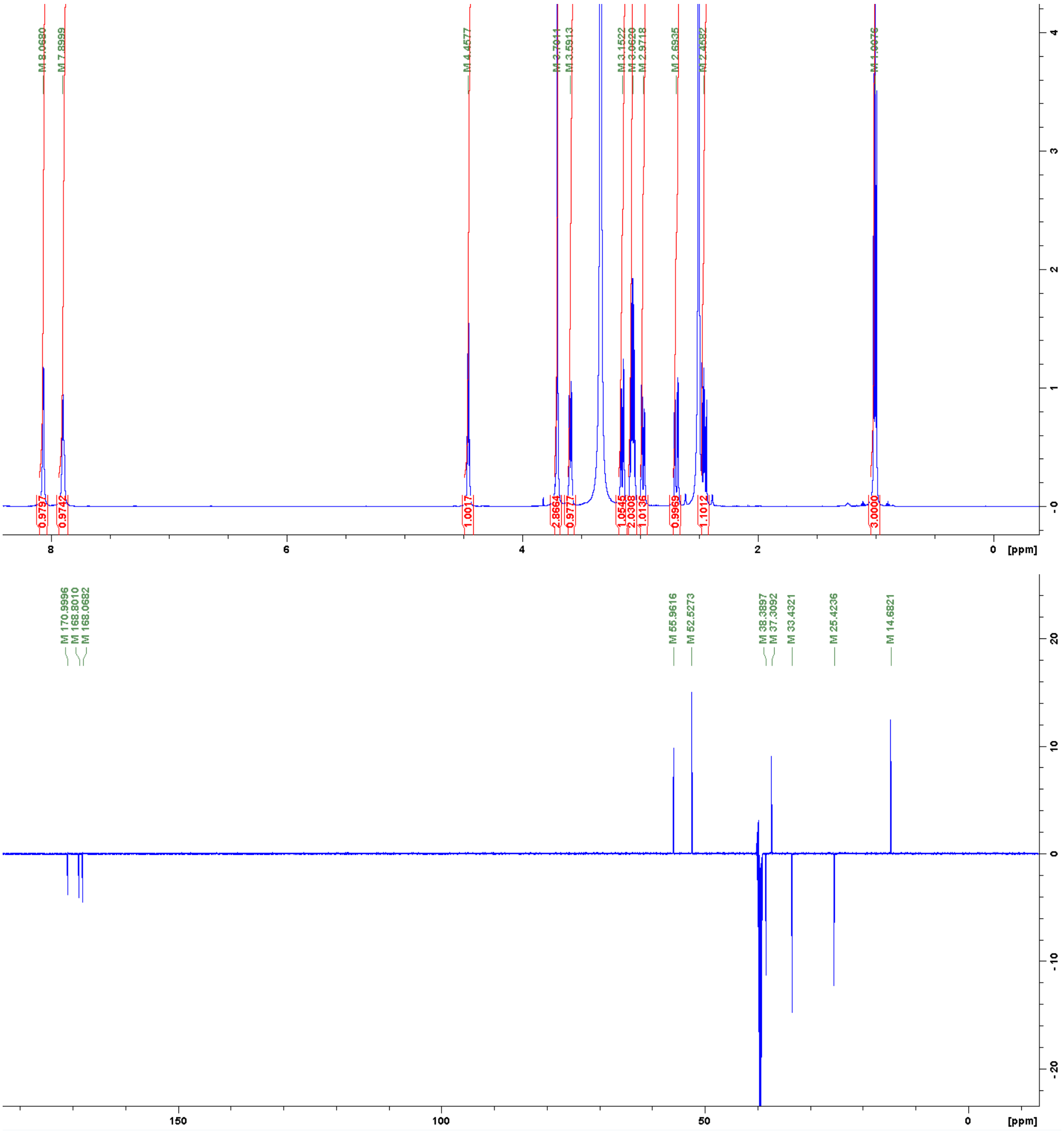

^{1}H and ^{13}C NMR spectra of **4.**

Oxaliplatin(IV)-maleimide-Cys-miniPEG-LARLLT conjugate (5a)

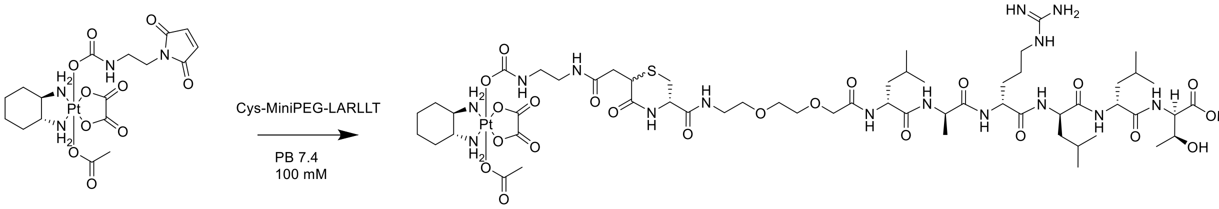

The compound was synthesized according to a modified procedure from reference [1]. (*OC*-6-33)-[(1*R*,2*R*)-Cyclohexane-1,2-diamine](2-(2,5-dioxo-2,5-dihydro-1*H*-pyrrol-1-yl)ethylcarbamato)oxalatoplatinum(IV) (20 mg, 31.27 µmol) and Cys-MiniPEG-LARLLT (42.7 mg, 34.4 µmol) were dissolved in 20 ml phosphate buffer (pH = 7.4, 100 mM) and stirred for 24 hours. The resulting mixture was lyophilized and purified via preparative HPLC (C_{18} X-Bridge, H_2O/ACN (both with 0.1% formic acid), 20–30% ACN over 30 minutes). Yield: 33.1 mg (67%) MS: calcd. for $[C_{57}H_{99}N_{15}O_{22}PtS+H^{+}+Na^{+}]^{2+}$ = 798.8133, found: 798.8217.

Oxaliplatin(IV)-maleimide-Ac-Cys-miniPEG-LARLLT conjugate (5b)

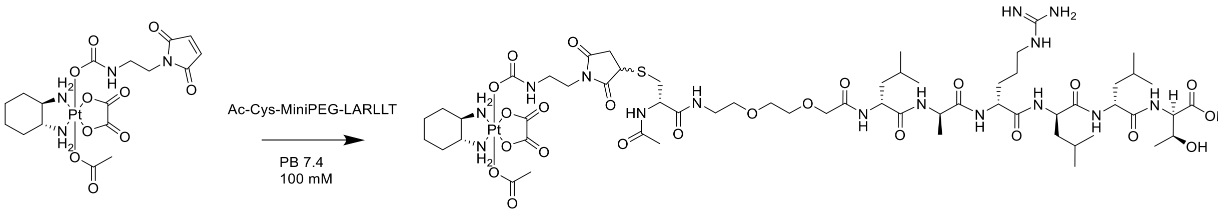

(*OC*-6-33)-[(1*R*,2*R*)-Cyclohexane-1,2-diamine](2-(2,5-dioxo-2,5-dihydro-1*H*-pyrrol-1-yl)ethylcarbamato)oxalatoplatinum(IV) (23.5 mg, 36.7 µmol) and Ac-Cys-MiniPEG-LARLLT (40 mg, 41 µmol) were dissolved in 9 ml phosphate buffer (pH = 7.4, 100 mM) and stirred for 2 hours. The resulting mixture was lyophilized and purified via preparative HPLC (C_{18} X-Bridge, H_2O/ACN (both with 0.1% formic acid), 20–30% ACN over 30 minutes). Yield: 32.2 mg (55%) MS: calcd. for $[C_{59}H_{101}N_{15}O_{23}PtS+H^{+}]^{+}$ = 1616.6641, found: 1616.6677; calcd. for $[C_{59}H_{101}N_{15}O_{23}PtS+H^{+}+Na^{+}]^{2+}$ = 819.8317, found: 819.8315.

Experimental details for X-ray diffraction and HPLC-MS

Crystallographic structure determination

The X-ray intensity data of compound **4** was measured on a Bruker D8 Venture diffractometer equipped with multilayer monochromators, Mo K/a INCOATEC micro focus sealed tubes and Oxford cryostream cooling device. The results were uploaded to the CCDC Number 2009419 (available online: http://www.ccdc.cam.ac.uk/conts/retrieving.html). Single crystals were placed with 38 mm distance to the detector, 1287 frames were measured over a time span of 60 s per frame and a frame angle of 0.6°. The structure was solved by *Charge Flipping*. Non-hydrogen atoms were refined with anisotropic displacement parameters. Hydrogen atoms were inserted at calculated positions and refined with a riding model. The following software was used:

Bruker SAINT software package[2] using a narrow-frame algorithm for frame integration, *SADABS*[3] for absorption correction, *OLEX2*[4] for structure solution, refinement, molecular diagrams and graphical user-interface, *Shelxle*[5] for refinement and graphical user-interface *SHELXS-2015*[6] for structure solution, *SHELXL-2015*[7] for refinement*,* *Platon*[8] for symmetry check. Crystal data, data collection parameters, and structure refinement details are given in Tables S1 and S2. Important bond length and angles are given in Table S3.

HPLC-MS studies

The HPLC part was carried out with a Waters Atlantis T3 C18 column (100 Å, 3 µm, 2.1 x 150 mm) and acetonitrile/Milli-Q water (both containing 0.1% formic acid) as eluents. A gradient from 1 to 99% acetonitrile over 20 minutes with 0.2 mL/min was applied. All samples (when taken from DSMO stock solution, final DMSO concentration did not exceed 5%) were dissolved in phosphate buffer (50 mM, pH=7.4), were incubated in the autosampler at 25°C and concentrations did not exceed 100 µM.

Figures and tables

Table S1: Sample and crystal data of **4**.

Radiation [Å]	MoKα (λ = 0.71073)	Z	4	Measurement method	\f and \w scans
Crystal habit	clear colourless plate	a [Å]	13.0078(9)		
Crystal size [mm^3]	0.1 × 0.03 × 0.005	b [Å]	4.9623(4)	Abs. correction type	multiscan
Empirical formula	C10H16N2O4S	c [Å]	28.828(2)	Abs. correction Tmin	0.6227
Formula weight [g/mol]	260.31	α [°]	90	Abs. correction Tmax	0.7460
Temperature [K]	100.0	β [°]	97.311(3)	Density (calculated) [g/cm^3]	1.389
Crystal system	monoclinic	γ [°]	90	Absorption coefficient [mm^{-1}]	0.338
Space group	P21	Volume [$Å^3$]	1845.7(2)	F (000) [e^-]	928.0

Table S2: Data collection and structure refinement of **4**.

2Θ range for data collection [°]	4.514 to 50.688	Index ranges		Goodness-of-fit on F^2	1.015
Reflections collected	46860	h	-15 ≤ h ≤ 15	Diff. peak and hole [$e^-Å^{-3}$]	0.27/-0.27
Data / restraints / parameters	6705/2/467	k	-5 ≤ k ≤ 5		
Refinement method	Charge Flipping	l	-34 ≤ l ≤ 34	Function minimized	Σ w (F_o^2 - F_c^2)^2
		all data	R1 = 0.0662, wR2 = 0.0836	Weighting scheme	where
		I>2σ(I)	R1 = 0.0428, wR2 = 0.076	w=1/[σ^2(Fo^2) + $(0.0474P)^2$ +0.3425P]	P=(F_o^2+2F_c^2)/3

Table S3: List of selected bond lengths and angles determined via the X-ray crystal structure of the transcyclization product **4.**

S1A- C5A	1.824(4) Å	C5A-S1A-C8A	97.1(2)°
S1A- C8A	1.790(4) Å	S1A-C5A-C6A	115.5(3)°
O2A- C6A	1.226(4) Å	O2A-C6A-N2A	121.4(4)°
N2A- C6A	1.345(5) Å	N2A-C6A-C5A	121.3 (4)°
N2A- C7A	1.451(5) Å	N2A-C7A-C8A	111.5(4)°
C5A- C6A	1.518(5) Å	C6A-C5A-S1A	115.7(3)°

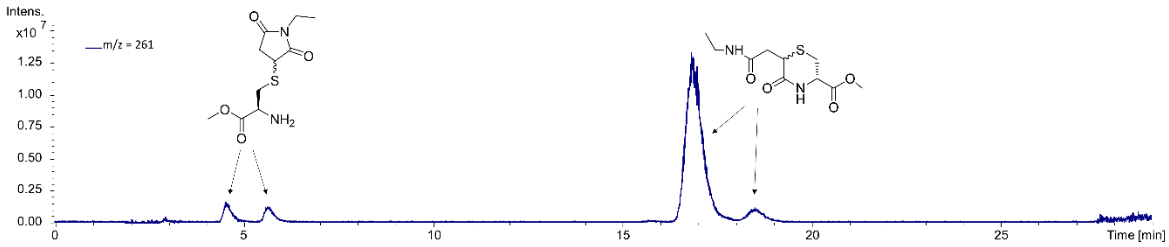

Figure S1. HPLC-MS EIC trace (*m/z* = 261) after 24 h incubation of 50 µM **1a** and **2** in PB (50 mM, pH 7.4, 37°C) with isocratic 99% H_2O/1% ACN (both with 0.1% formic acid). Under these conditions the second diastereomer of **4** is clearly visible at 18.5 min.

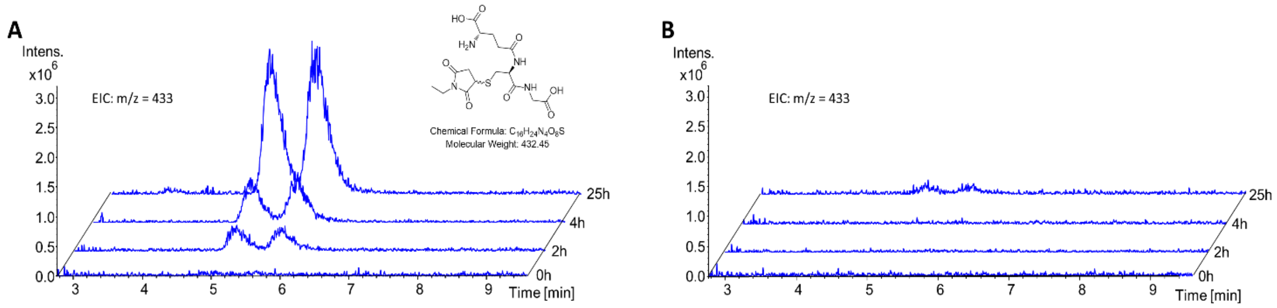

Figure S2. HPLC-MS EIC trace (m/z = 433) of the N-ethylmaleimide-GSH adduct (rt = 5 and 5.8 min). Incubation of 50 µM **3b** (A) and **4** (B) in PB (100 mM, pH = 7.4) with a 10-fold excess of reduced GSH at 25°C over 25 h.

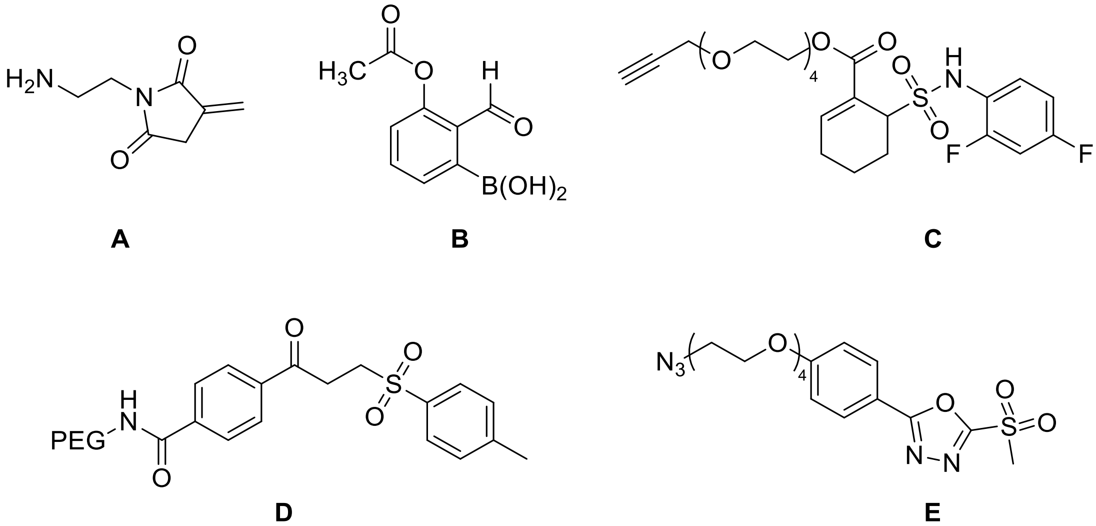

Figure S3. Overview on HSA binding alternatives to common maleimides: **A** exo-cyclic maleimide; **B** 2-formylphenylboronic acid; **C** TAK-242 derivative targeting Lys64; **D** Sulfone derivative used for exogenous HSA coupling and **E** phenyl-oxadiazole-sulfone moiety for Cys-34 targeting.

References

[1] Mayr, J., Hager, S., Koblmüller, B., Klose, M.H.M., Holste, K., Fischer, B., Pelivan, K., Berger, W., Heffeter, P., Kowol, C. R., Keppler, B. K. EGFR-targeting peptide-coupled platinum(IV) complexes. J. Biol. Inorg. Chem. 2017, 22, 591–603.

[2] Bruker SAINT v8.38B Copyright © 2005-2019 Bruker AXS

[3] Sheldrick, G. M. (1996). *SADABS.* University of Göttingen, Germany.

[4] Dolomanov, O.V., Bourhis, L.J., Gildea, R.J, Howard, J.A.K. & Puschmann, H. , OLEX2, (2009), J. Appl. Cryst. 42, 339-341

[5] C. B. Huebschle, G. M. Sheldrick and B. Dittrich, ShelXle: a Qt graphical user interface for SHELXL, J. Appl. Cryst., 44, (2011) 1281-1284

[6] Sheldrick, G. M. (2015). *SHELXS v 2016/4* University of Göttingen, Germany.

[7] Sheldrick, G. M. (2015). *SHELXL v 2016/4* University of Göttingen, Germany.

[8] A. L. Spek, Acta Cryst. 2009, D65, 148-155.
